# Inferior Longitudinal Fasciculus’ Role in Visual Processing and Language Comprehension: A Combined MEG-DTI Study

**DOI:** 10.3389/fnins.2019.00875

**Published:** 2019-08-23

**Authors:** Jiwon Shin, Jared Rowley, Rasheda Chowdhury, Pierre Jolicoeur, Denise Klein, Christophe Grova, Pedro Rosa-Neto, Eliane Kobayashi

**Affiliations:** ^1^Montreal Neurological Institute and Hospital, McGill University, Montreal, QC, Canada; ^2^Translational Neuroimaging Laboratory, McGill University Research Centre for Studies in Aging, McGill University, Montreal, QC, Canada; ^3^Multimodal Functional Imaging Lab, Department of Biomedical Engineering, McGill University, Montreal, QC, Canada; ^4^Centre de Recherche en Neuropsychologie et Cognition (CERNEC), Département de Psychologie, Université de Montréal, Montreal, QC, Canada; ^5^Cognitive Neuroscience Unit, Montreal Neurological Institute, Neurology and Neurosurgery, McGill University, Montreal, QC, Canada; ^6^Multimodal Functional Imaging Lab, PERFORM Centre, Department of Physics, Concordia University, Montreal, QC, Canada

**Keywords:** diffusion tensor imaging, language comprehension, magnetoencephalography, ventral language pathway, white matter

## Abstract

The inferior longitudinal fasciculus (ILF) is a white matter tract that connects the occipital and the temporal lobes. ILF abnormalities have been associated with deficits in visual processing and language comprehension in dementia patients, thus suggesting that its integrity is important for semantic processing. However, it remains elusive whether ILF microstructural organization *per se* impacts the visual semantic processing efficiency in the healthy brain. The present study aims to investigate whether there is an association between ILF’s microstructural organization and visual semantic processing at the individual level. We hypothesized that the efficiency of visual semantic processing positively correlates with the degree of anisotropy of the ILF. We studied 10 healthy right-handed subjects. We determined fractional anisotropy (FA) of the ILF using diffusion tensor imaging (DTI). We extracted N400m latency and amplitude from magnetoencephalography (MEG) signals during a visual semantic decision task. N400m and mean FA of the ILF were left lateralized with the higher FA value in the left hemisphere. Inter-individual analysis showed that FA of the ILF negatively correlated with the N400m latency and amplitude, which suggests that high ILF anisotropy is associated with more efficient semantic processing. In summary, our findings provide supporting evidence for a role of the ILF in language comprehension.

## Introduction

The inferior longitudinal fasciculus (ILF) is a major ventral associative bundle that connects and transfers information between the occipital and the temporal lobes. Due to its location, it has been speculated that the ILF allows fast transfer of visual information (Mishkin et al., 1983; Catani et al., 2003; Catani and Thiebaut de Schotten, 2008). Recent findings suggest that, indeed, the ILF plays a role in object recognition (Ortibus et al., 2012) and face processing (Taddei et al., 2012).

Semantic processing is part of language comprehension, during which the meaning of a presented word is retrieved and interacts with concepts that have associated meanings (Quillian, 1967). Although there is evidence for the ILF’s role in both visual processing and in language comprehension, it remains debatable whether the ILF influences visual semantic processing, which takes place during silent reading.

Pathways determined by white matter tracts can be investigated *in vivo* using Diffusion Tensor Imaging (DTI), which non-invasively measures three-dimensional diffusion properties of water molecules (Mori and Zhang, 2006; Jones, 2008; Behrens and Jbabdi, 2009). White matter fractional anisotropy (FA) is a DTI outcome measure that expresses the degree of anisotropy of diffusion, which is restricted by the microarchitectural organization imposed by axon bundles and their respective myelin sheaths. FA can be used to infer the degree of tissue microstructural organization and connectivity as well as white mater integrity (Pierpaoli and Basser, 1996).

In a DTI study involving a sound-to-word learning task, the ILF has been suggested to mediate acoustic and semantic processing, as the FA of the ventral language pathway positively correlated with auditory semantic processing ability (Wong et al., 2011). In neurological populations, an association between ILF FA and cognition has been reported in Alzheimer’s disease (Zimny et al., 2012) and semantic dementia (Powers et al., 2013). Patients with semantic dementia have reduced left ILF FA and radial diffusivity, which reflected changes in the white matter organization and suggested microstructural reorganization (Whitwell et al., 2010).

During visual semantic processing, a sequence of events is identifiable through neurophysiology. Event-related potentials (ERPs) using electroencephalography and event-related fields (ERFs) using magnetoencephalography (MEG) recordings reveal several characteristic components of visual semantic processing. In MEG, visual input triggers a series of early components, including the N100m, which is strongly influenced by physical properties of the stimuli. N100m is followed by M170, known to reflect the category and task relevance of stimuli. N100m and M170 are followed by later ERFs that reflect integration with context and meaning, such as the N400m (Kutas, 1993; Billingsley-Marshall et al., 2007; Service et al., 2007; Lau et al., 2009).

N400m has been identified as an index of lexical-semantic processing, associated with lexical retrieval and language comprehension (Kutas and Hillyard, 1980; Marinkovic et al., 2003; Lau et al., 2009; Kutas and Federmeier, 2011). N400m is localized in the left superior and middle temporal gyri in right-handed subjects around 400 ms after the visual stimulus presentation using MEG (Helenius et al., 1998; Halgren et al., 2002; Billingsley-Marshall et al., 2007; Service et al., 2007; Lau et al., 2008).

In this study, we investigated the existence and degree of an association between mean FA of the ILF and visual semantic processing measured by N400m in right-handed healthy subjects, who are highly likely to have left hemisphere language dominance (Knecht et al., 2000). We hypothesized that visual semantic processing efficiency is associated with the microstructural integrity of the ILF in the individual level. In order to address this hypothesis, we investigated the correlation between the mean FA of the ILF through DTI analysis and N400m latency and amplitude through MEG mapping.

## Materials and Methods

### Ethical Aspects

This study was carried out in accordance with the recommendations of the Montreal Neurological Institute Research Ethics Board with written informed consent from all subjects. All subjects gave written informed consent in accordance with the Declaration of Helsinki. The protocol was approved by the Montreal Neurological Institute Research Ethics Board.

### Subjects

A total of 19 healthy right-handed fluent English-speaking subjects answered to our advertisement at campus and volunteered to participate in this study, which comprised an MRI-DTI acquisition and a MEG session (8 female, age 18–40 years, mean: 26.2 ± 5.8 years). Handedness was determined using the Edinburgh Handedness Inventory. Left-handed subjects were excluded, as they might have atypical language representation (Duffau et al., 2008). All subjects had normal vision or normal vision through corrective lenses.

Six subjects were excluded from further MEG data analysis due to excessive motion (*N* = 2), excessive blinking (*N* = 2), incomplete acquisition due to technical problems (*N* = 1), and suboptimal head position, which interfered with the subject ability to see the experimental stimuli (*N* = 1).

From the remaining 13 subjects that underwent analysis of MEG signals, three were further excluded based on quality of individual N400m (see “MEG Analysis” section below for more detailed information). In brief, a clear peak between 300 ms and 500 ms with a higher than 1.5 in the MEG signals was required for correlation with DTI data. Thus, this study reports results from 10 subjects: 5 female (age 18–36 years, mean 23 ± 5.5 years) and 5 male (age 19–36 years, mean 27.6 ± 6 years). Further details of inclusion and exclusion criteria for this study are explained below.

### MEG Data Acquisition

Magnetoencephalography signal recording took place at University of Montreal using a CTF system (VSM MedTech Ltd., Canada), equipped with 275 axial gradiometers. We recorded MEG signals at 1200 Hz sampling rate. We monitored eye movements and blinks using vertical and horizontal electro-oculogram electrodes, and cardiac artifacts through electrocardiogram monitoring. As part of a larger MEG study, the MEG mapping protocol reported here included a lexical-semantic categorization task. This semantic task was designed for subjects to actively process stimuli at the level of meaning and to elicit brain responses associated with semantic processing of a visually presented word.

We visually presented 240 category-exemplar word pairs (example: FLOWER/ROSE) projected on a screen, with trials divided in 4 test blocks of 60 trials. Category and exemplar words were divided into two equiprobable conditions: related pairs (example: FLOWER/ROSE) and unrelated pairs (example: FLOWER/SOCCER). The subject had to decide whether or not the exemplar word named a member of the category word. The exemplar word in the related condition was expected to elicit a smaller N400m due to easier lexical and semantic integration with the category concept as compared with the unrelated condition. Each category word was shown for 500 ms, followed by the exemplar word, which was presented until the subject responded.

The subject had to press one button with the thumb of the left or right hand, within 2 s of target word presentation. Beyond 2 s, the response was considered late, the experiment continued with the next word pair and that trial was excluded from the analysis. Instruction on which button to press was given at the beginning of each test block. After two blocks, subjects were instructed to reverse the sides of the response. A feedback signal was displayed after the subject’s button press or after 2 s of the target presentation if he/she did not make a button press. The feedback was presented visually at the center of the screen for 500 ms: “+” for correct, “−” for wrong, and “l” for late responses.

The list of items was constructed from a list of 12 high-frequency words in each of 17 different categories (Van Overschelde et al., 2004), which excluded homonyms and compound words. Categories consisted of animals, birds, body parts, carpenter’s tools, clothing, colors, countries, flowers, fruits, furniture, insects, and musical instruments. The frequency of appearance of each word was controlled to minimize the effects of priming that can result in less semantic memory searching, less cognitive processing, and smaller N400m amplitudes (Kutas and Federmeier, 2000; Matsumoto et al., 2005; Federmeier, 2007; Lau et al., 2009).

### MEG Analysis

We processed MEG signals using Data-Editor software (CTF, VSM MedTech Ltd., Canada). Signals underwent sub-sampling at 600 Hz, third order gradient correction, offset removal, band-pass filtering (0.3–40 Hz), and removal of epochs containing motion artifact, eye blinks and large eye movements.

For each trial, an epoch was segmented and baseline-corrected, containing signals from −200 ms to +700 ms in relation to the onset of the second word presentation in each trial (i.e., the exemplar). We excluded errors and late responses. Trials with correct responses were used to compute an average signal dataset for “related” and “unrelated” conditions. To isolate brain activity specifically related to the difference in processing for semantically related versus unrelated exemplars, we subtracted signals in the “related” condition average from signals in the “unrelated” condition average.

**FIGURE 1:**
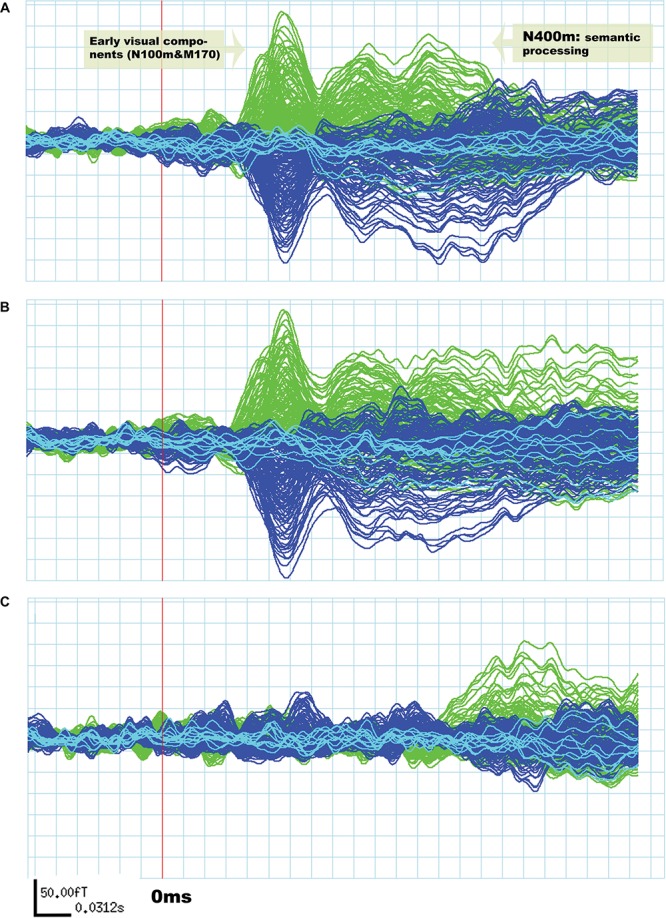
Evoked response field from MEG signals related to silent reading of word pairs in a single subject. The signals in green are derived from the left hemisphere sensors and the signals in blue are from the right hemisphere. In **(A)** the “unrelated” condition and in **(B)** the “related” condition. Early visual components (N100m and M170) are not significantly different between “unrelated” and “related” conditions. The signal in **(C)** is a subtraction of the “unrelated” condition from the “related” condition.

Although the amplitude of N400m was expected to be larger in the “unrelated” condition than in the “related,” as found in previous research (Kutas and Federmeier, 2000; Matsumoto et al., 2005; Federmeier, 2007; Lau et al., 2009), differences across these conditions should be minimal for early visual components (N100m and M170) (Tarkiainen et al., 1999; Wydell et al., 2003; Pammer et al., 2004; Maurer et al., 2008). The results agreed with these expectations, as can be seen in Figure 1.

**FIGURE 2:**
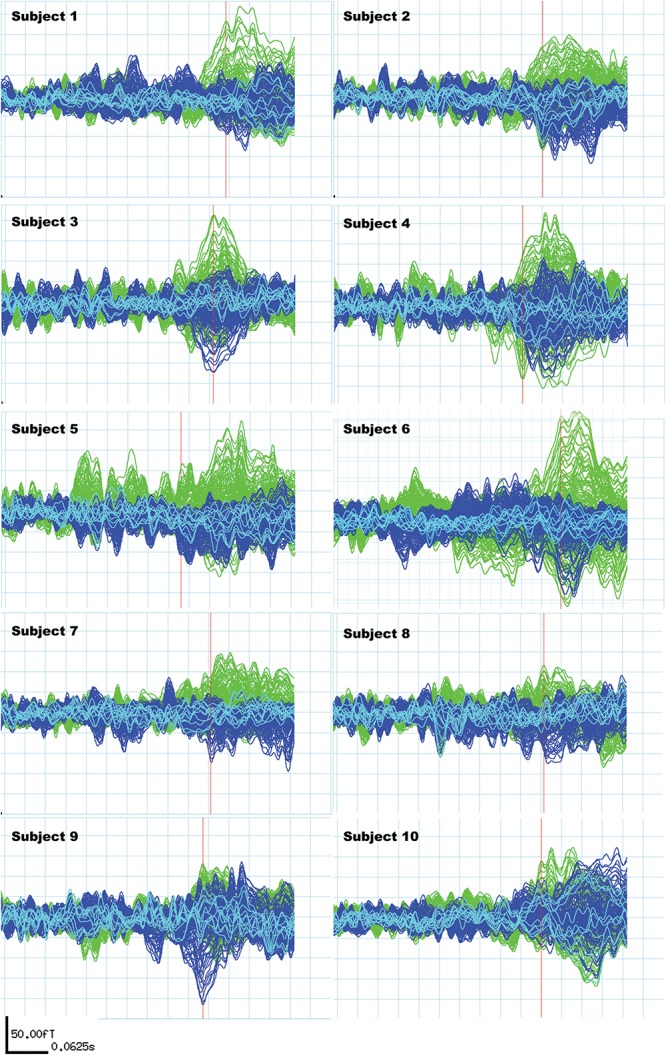
Individual N400m signals of all 10 subjects. These signals are subtractions of the “unrelated” condition average from the “related” condition average to reflect the difference in N400m response only. The green signals represent activities recorded from the left hemisphere and the blue signals represent activities recorded from the right hemisphere. Light blue signals derive from midline sensors. The red bar locates the time point used to measure N400m latency and amplitude, which is the first peak within the N400m time window (300–500 ms) with a signal-to-noise ratio higher than 1.5 for each subject.

Subjects included in the study fulfilled the following MEG signal criteria: N400m response in the subtracted signal present in a time window from 300 to 500 ms (Service et al., 2007; Lau et al., 2009), with a signal-to-noise ratio (SNR) of 1.5 and above compared to the baseline (Figure 2). The SNR was determined comparing the amplitude and the peak amplitude in fT in the baseline signals (defined as the epoch between −200 and 0 ms in relation to the stimulus presentation).

Latency of N400m was assessed at the first peak observed between 300 and 500 ms of the extracted N400m signal, corresponding to the earliest time point within the ascending slope of the N400m peak showing a peak of 1.5 SNR as compared to the baseline. This early time point was selected in order to allow better differentiation analysis of latency, as opposed to the maximum peak. At that point, we also measured amplitude as compared to the baseline.

We performed a supplementary analysis to assess the specificity of the relationship between ILF FA and semantic processing. In order to rule out whether the ILF could rather have a diffuse non-specific association with cognitive functions, we evaluated the auditory mismatch field (MMF) recorded simultaneously with N400m and other ERFs as part of a multifunctional MEG study, with a interleaved design ensuring no interaction between different stimuli. A general correlation with ILF’s FA might be expected, for example, if FA in the ILF reflects a global property of the integrity of white matter, in which case a correlation would not support specific measures related to the occipital-temporal connectivity.

Mismatch field is an ERF that reflects early cognitive auditory processing and does not involve semantic processing. It is activated at 150–250 ms after onset of a deviant stimulus in the primary and secondary auditory cortices and right inferior frontal gyrus (Hari et al., 1984). During the same MEG recording session, non-lexical sounds (pure tones, at 1000 and 1300 Hz) were presented binaurally through earphones in an oddball paradigm in a 1:7 ratio; rare deviant stimuli were interspersed amongst a series of frequent standard stimuli. Since the MMF is evoked by the infrequent change in pitch, we extracted MMF responses by subtracting the signals in the deviant condition average from the signals in the frequent condition (Jacobson, 1994; Alho, 1995). There was no task associated with this stimulus presentation. In order to determine whether the effect of the ILF on visual semantic processing is due to a broad effect in cognitive processing, we examined the correlation between the mean FA of the ILF and MMF latency using a one-tailed Pearson’s correlation. We compared it to the correlation between the mean FA of the ILF and N400m latency. Since MMF does not involve areas connected through the ILF pathways, we expected to find no correlation with the ILF’s FA.

### Behavioral Data

We evaluated each subject’s mean reaction time and accuracy. The reaction time corresponded to the time it took from the onset of the exemplar word to the subject’s button press response. We calculated the accuracy of performance in the semantic decision task by counting the correctly responded trials over the total number of trials in the 4 test blocks (240 trials). Late responses were counted as errors.

### MRI Data Acquisition

Magnetic resonance imaging (MRI) data were acquired at the Montreal Neurological Institute Brain Imaging Center on a Siemens Tim Trio 3T scanner using a 32-channel head coil. Head cushions were placed within the head coil to minimize the subject’s head motion. Anatomical acquisition consisted of a T1-weighted magnetization prepared rapid gradient echo (MPRAGE) sequence, with 1 mm isotropic three-dimensional acquisition with the following parameters: 192 sagittal slices, 256 × 256 matrix, TE = 2.98 ms, TR = 2.3 s, flip angle 9°. Diffusion MR images were obtained subsequently from 99 independent non-collinear directions (*b*-value of 1000 s/mm^2^) and ten *b* = 0 images with no diffusion gradients which provided a reference for image processing.

### DTI Analysis

We processed the DTI data using Functional Magnetic Resonance Imaging of the Brain (FMRIB) Software Library Diffusion Toolkit (FSL-FDT) (Smith et al., 2004; Jenkinson et al., 2012). We applied Eddy Current Correction tool for motion correction – DTI images were corrected to minimize distortions such as stretch, shears, and simple head motion using affine registration to a resting state volume (b_0 image) as reference. We used Brain Extraction Tool to exclude non-brain tissues from the whole head images (Smith, 2002).

Diffusion tensors at each voxel were calculated using DTIFIT in order to generate FA maps. DTIFIT computed the diffusion tensor eigenvalues that described the diffusion coefficients in the primary, secondary, and tertiary diffusion directions (eigenvectors). In order to reconstruct the ILF in each subject, we used the ILF mask from a DTI-based white-matter atlas from Johns Hopkins University (Mori et al., 2005; Wakana et al., 2007; Hua et al., 2008)^1^. This atlas is based on white matter tracts identified probabilistically by averaging 28 normal subjects’ deterministic tractography results. Each tractography involved multiple regions of interest covering the different parts of the ILF, which were defined based on existing anatomical knowledge. The reconstructed tracts were transformed into standard space and averaged to generate probabilistic maps for each major white matter tract. These maps show the probability of the presence of a tract at each voxel in Montreal Neurological Institute (MNI) space with any voxel showing a probability greater than 0.

**FIGURE 3:**
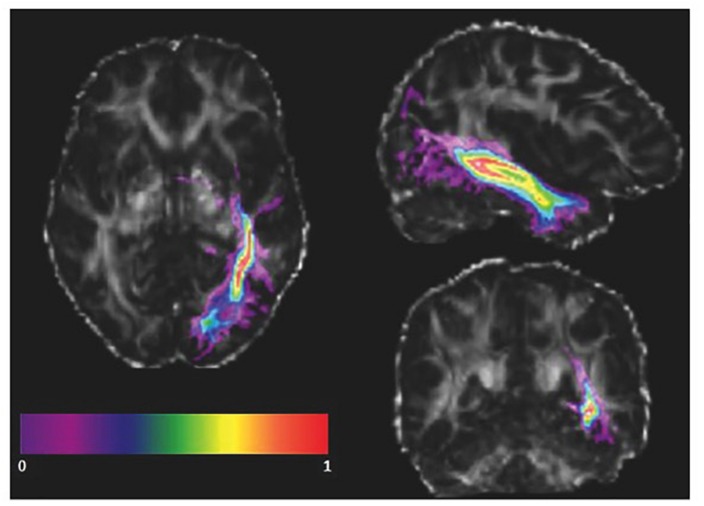
Registered atlas-based ILF mask of a single subject. The probabilistic left ILF mask obtained from the JHU atlas (color) is co-registered with a subject’s FA map in standard space in radiological orientation. The masked brain areas were included in the weighted mean FA calculation.

As the ILF might vary amongst healthy subjects in the native space, the advantage of using the ILF mask from an atlas instead of manually defining regions of interest for each subject is that the number of voxels included in the ILF masks is equal across subjects. Furthermore, FA values within the ILF mask into native space and stereotaxic space were correlated. We transformed our subjects’ FA maps into the standard space and co-registered them with the ILF mask from the atlas (Figure 3). We visually inspected the co-registration quality, and subsequently applied the ILF mask as a volume of interest (VOI). In order to avoid the inclusion of gray matter voxels present in the vicinity of the defined ILF VOI, we calculated a weighted average of the FA values of each voxel included in the ILF VOI in both the left and right hemispheres. Thus, instead of averaging every voxel of the ILF mask equally, we weighted each voxel based on the probability of the voxel being part of the ILF. The weighted average was computed as:

$$\frac{1}{\sum_{\upsilon }\mathrm{Prob}\left(\upsilon \in \mathrm{ILF}\right)}\sum_{\upsilon }\mathrm{Prob}\left(\upsilon \in \mathrm{ILF}\right)\cdot \mathrm{FA}\left(\upsilon \right)$$

A lateralization index was computed as [2(FA_*left*_ − FA_*right*_)]/(FA_*left*_ + FA_*right*_).

### Statistical Analysis

We used a one-tailed paired *t*-test to confirm whether the mean FA in the left hemisphere was greater than in the right hemisphere, as previously described in the literature (Verhoeven et al., 2010; Thiebaut de Schotten et al., 2011; Menjot de Champfleur et al., 2012). In order to examine inter-subject differences in ILF anisotropy and in efficiency of visual semantic processing, we applied a one-tailed Pearson’s correlation on the mean FA of the ILF in the left hemisphere and N400m latency/amplitude for each subject. This allowed us to examine if efficient semantic processing correlates with higher anisotropy of the ILF. We assessed correlation between the mean FA values of the right ILF and N400m indices in order to determine if a correlation would be specific to the left ILF, considering the left hemispheric dominance for language.

Considering that our results could be biased by the limiting sampling size of our population, in addition to standard linear regression analysis, we also performed a non-parametric Spearman’s rank correlation coefficient test (Spearman’s rho and *p*-value of the one and two tailed statistical tests are provided).

## Results

**FIGURE 4:**
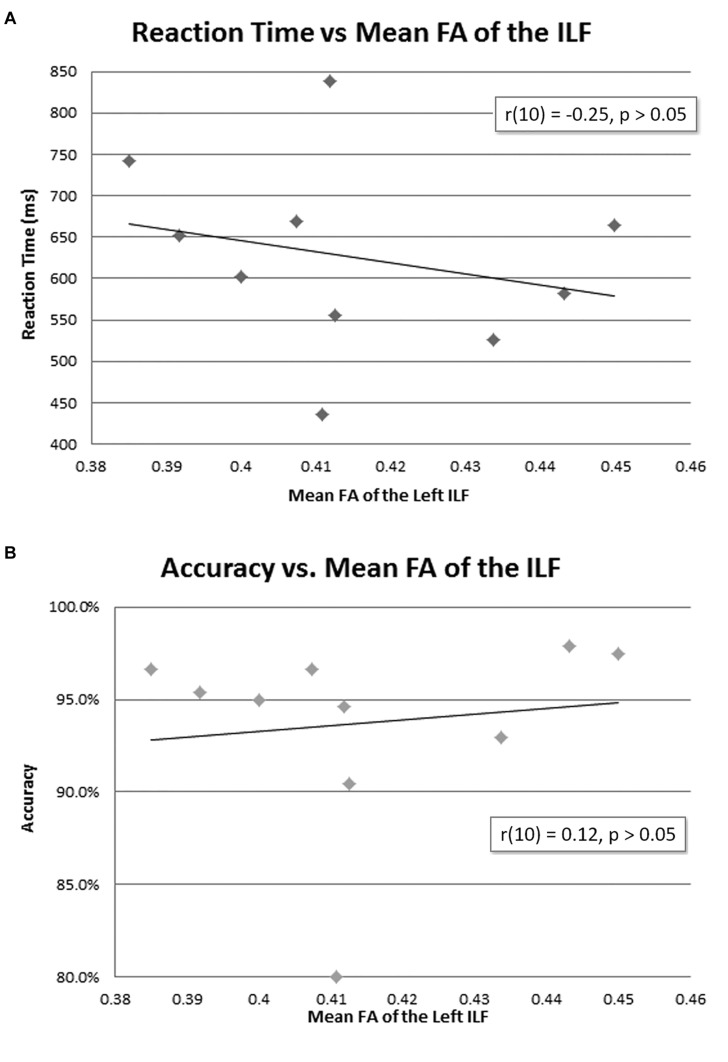
Behavioral data and mean FA of the left ILF. No correlation was found between mean FA of the left ILF and reaction time **(A)** or accuracy **(B)**.

The mean reaction time was 627 ms (Table 1). There was no correlation between mean FA of the left ILF and reaction time [*r*(10) = −0.25, *p* = 0.23, with a *R*^2^ = 0.06] or accuracy [*r*(10) = 0.12, *p* = 0.35, with a *R*^2^ = 0.02] (Figure 4).

**TABLE 1:** Summary of behavioral data.

	**Behavioral Data**		**Mean FA (ILF)**		**N400m**	
**Subject**	**Reaction time (ms)**	**Accuracy (%)**	**Left**	**Right**	**Latency (ms)**	**Amplitude (fT)**
1	742	96.7	0.3850	0.3502	490	195
2	581	97.9	0.4431	0.4204	440	135
3	652	95.4	0.3917	0.3850	450	215
4	437	80.0	0.4108	0.3878	378	140
5	665	97.5	0.4499	0.4443	350	115
6	601	95.0	0.4000	0.3906	497	265
7	669	96.7	0.4073	0.4005	445	120
8	838	94.6	0.4119	0.3992	447	115
9	556	90.4	0.4126	0.4051	420	210
10	527	92.9	0.4337	0.4425	438	145
Mean	626	93.7	0.4146	0.4026	436	166
Standard deviation	113	5.3	0.0214	0.0281	45	52

*Reaction time, mean time from target word presentation to button press; Accuracy, correct responses; ms, millisecond; fT, femtotesla; FA, fractional anisotropy; ILF, inferior longitudinal fasciculus.*

N400m ERF was lateralized in the left hemisphere in all subjects. Four subjects showed similar topography in the right hemisphere, but weaker in amplitude compared to the left hemisphere (see below). The mean N400m latency was 436 ms and the mean N400m peak amplitude of all channels was 166 fT (Table 1). Figure 2 illustrates the extracted N400m signal for each subject, which contains an N400m peak with a SNR ratio higher than 1.5 in the N400m time window (300–500 ms).

**FIGURE 5:**
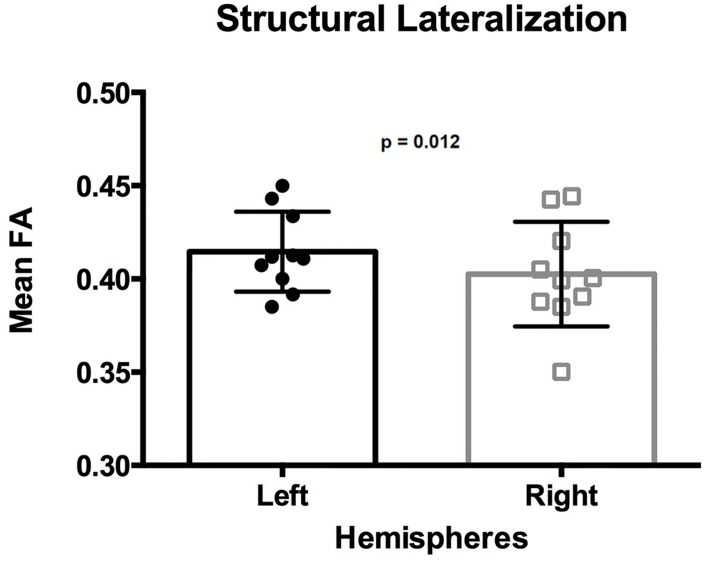
Lateralization of the ILF determined by the mean FA. Each dot and square represents one individual mean FA value. Error bars represent standard deviation. The mean FA of the left ILF is significantly higher than that from the right ILF (*p* = 0.012).

The mean FA of the ILF was 0.415 in the left hemisphere and 0.403 in the right hemisphere (Table 1), with a mean lateralization index of 0.015 (SD 0.016; range −0.010 to ∼ 0.047). The mean FA of the ILF was significantly higher in the left hemisphere than in the right hemisphere, [one-tailed paired *t*-test *t*(9) = 3.154, *p* = 0.012] (Figure 5).

**FIGURE 6:**
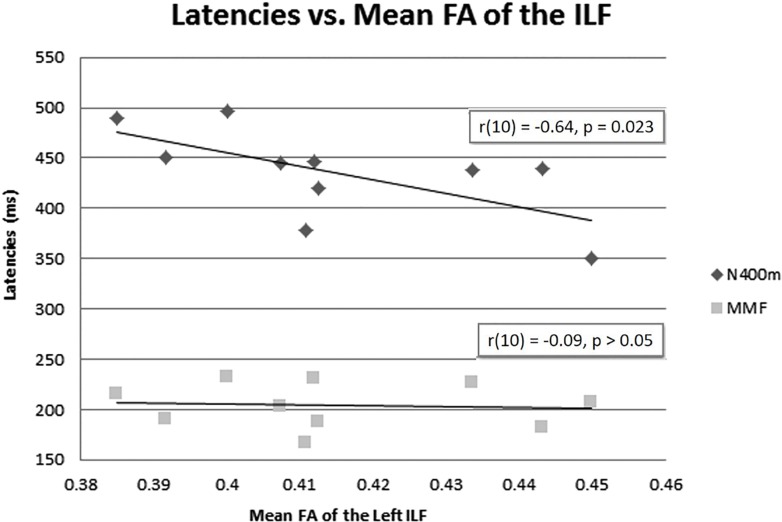
N400m and MMF latencies and mean FA of the left ILF. A negative correlation between N400m peak latency and mean FA of the left ILF has been identified [*r*(10) = –0.64, *p* = 0.023, *R*^2^ = 0.41]. No correlation was found between MMF and mean FA of the left ILF or between N400m latency and mean FA of the right ILF.

**FIGURE 7:**
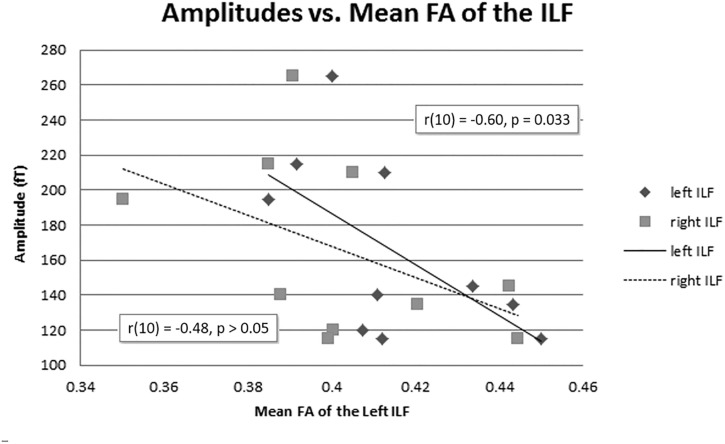
N400m amplitudes and mean FA of the ILF. A negative correlation between N400m peak amplitude and mean FA of the left ILF has been identified [*r*(10) = –0.60, *p* = 0.033, *R*^2^ = 0.36]. The right ILF did not show a significant correlation with N400m peak amplitude (*p* = 0.080).

The mean FA of the left ILF and N400m latency showed a significant negative correlation, *r*(10) = −0.64, *p* = 0.023, with a *R*^2^ = 0.41 (Figure 6), rho = −0.7697; *p* = 0.0137; 1 tailed *p* = 0.0068. There was also a negative correlation between the mean FA of the left ILF and N400m amplitude, *r*(10) = −0.60, *p* = 0.033, with a *R*^2^ = 0.36 (Figure 7), (rho = −0.5654; *p* = 0.0885; 1 tailed *p* = 0.0443).

The mean FA of the right ILF negatively correlated with N400m latency, *r*(10) = −0.57, *p* = 0.04, with a *R*^2^ = 0.33; (rho = −0.6485; *p* = 0.0490; 1 tailed *p* = 0.0245). However, the mean FA of the right ILF did not show any significant correlation with N400m amplitude, *r*(10) = −0.48, *p* = 0.080, with a *R*^2^ = 0.23; (rho = −0.4863; *p* = 0.1541; 1 tailed *p* = 0.0770).

We found no correlation between the mean FA of the left ILF and the latency of MMF [*r*(10) = −0.09, *p* = 0.49, *R*^2^ = 0.00] (Figure 6), (rho = −0.1879; *p* = 0.6076; 1 tailed *p* = 0.3038). This suggests that the association of higher FA of the left ILF with a shorter latency of N400m response was less likely a global phenomenon or effect.

To clarify if age could be identified in our subjects, we have performed Spearman’s rank correlation coefficient and found no significant effect on left FA (rho = −0.2188; *p* = 0.5436; 1 tailed *p* = 0.2718), right FA (rho = −0.3404; *p* = 0.3358; 1 tailed *p* = 0.1679), N400 Latency (rho = 0.1581; *p* = 0.6628; 1 tailed *p* = 0.3314) and N400 Amplitude (rho = 0.0061; *p* = 0.9867, 1 tailed *p* = 0.4933). These results could suggest that the age range of our subjects was too limited to exhibit any known effect of age that could have biased the results.

## Discussion

This study combines DTI and MEG techniques to investigate correlations between white matter microstructural integrity of temporal-occipital connections and visual semantic processing efficiency. We found an association between ILF anisotropy and the efficiency of visual semantic processing measured by N400m. N400m latency and amplitude negatively correlated with the mean FA of the left ILF, which suggests that efficient visual semantic processing is associated with high anisotropy of the ILF. No correlation was found with auditory processing, a task not related to ILF connectivity, ruling out a more global effect.

The mean FA of the right ILF did not show a significant correlation with N400m amplitude, but did with N400m latency with less correlation than the left ILF mean FA, which barely passed the critical value. This suggests that although the ILF is homologous in left and right hemispheres and high symmetry is expected, we were able to examine that the structural differences expressed by anisotropy exist and that this difference is also represented as the functional difference, assuring the specificity of left-lateralization of visual semantic processing assessed by N400m and anisotropy. The relationship between function and structure might reflect individual ability and efficiency of cognitive functions, because the white matter tracts connect and transmit information between brain areas in a functional network (Johansen-Berg, 2010).

Our results support previously reported findings on the ILF’s involvement in language comprehension, which includes semantic processing (Mandonnet et al., 2007; Saur et al., 2008; Wong et al., 2011). Wong demonstrated that anisotropy of the ventral language pathway, including the ILF, positively correlated with performance in sound-to-word learning. The task involved hearing and learning foreign phonetic contrasts for signaling word meaning, which tests acoustic processing and semantic processing. Wong’s study suggested that this language comprehension function of the ventral language stream is mainly subserved by the ILF (Wong et al., 2011).

We extend this finding further to show a role for the ILF in semantic processing in the visual modality. The expected negative correlation between reaction time and mean ILF’s anisotropy was refuted in the present study, suggesting that the link between FA and cortical responses cannot be extrapolated to response reaction times. Similarly, the accuracy of a participant’s response in the semantic decision task was independent from the ILF’s FA. In fact, the absence of association between response accuracy and ILF’s FA could be explained by a ceiling effect due to high accuracy (all above 80% with 93.7% mean group accuracy) in performance, which confirmed the participants’ comprehension, attention and engagement during the task. In order to measure the N400m latency, we considered the first peak with an SNR higher than 1.5 in relation to the baseline within the N400m time window, in accordance with the approaches used in other studies observing the N400m and N400 latency. The peak latency in the subtracted condition ERF is measured using a grand-average signal containing a single peak which is not dispersed and has a high SNR compared to a single-subject signal (Ardal et al., 1990; Grillon et al., 1991; Kutas and Iragui, 1998; Olichney et al., 2002; Moreno and Kutas, 2005). However, in our study, the estimating method may be prone to observer’s bias, because latency peak can be ambiguous at a single-subject level. The negative correlation between N400m values and ILF anisotropy corroborates the current view on N400m latency and amplitude as an index for cognitive processing ability and efficiency (Helenius et al., 1998; Kitade et al., 1999). N400m latency, the speed at which the meaning of a word can be activated, is often studied as an index of cognitive processing ability and efficiency (Helenius et al., 1998). The latency and amplitude of N400m are larger in aphasic patients as compared to healthy subjects (Kitade et al., 1999). The reduced latency of N400m may be explained by the transfer efficiency in the semantic processing network – the shorter the latency, the more efficiently information is transferred from the occipital lobe to the temporal lobe. Also, the amplitude negatively has been shown to reflect facilitated processing of semantic information, suggesting the N400m amplitude as an index for processing efficiency (Kitade et al., 1999; Lau et al., 2009; Taylor et al., 2011; van den Brink et al., 2012). Our data suggests that inter-individual differences in white matter microstructure are associated with individual ability and efficiency of cognitive functions (Passingham et al., 2002; Johansen-Berg, 2010). Various factors such as semantic category of a word, level of engagement in processing, and frequency of a word, contribute to N400m amplitude changes. The degree of such influence may vary at the individual level (Kiefer, 2001; Van Petten, 2006). On the other hand, N400m latency does not vary as much. Only a few factors such as aging (i.e., longer latency in the aged individuals), neurological or psychiatric conditions, such as schizophrenia and Alzheimer’s disease (i.e., longer latency in neurological and psychiatric patients), and language proficiency (i.e., latency decreases with years of language experience and it increases with age of exposure) have been associated with N400m latency (Ardal et al., 1990; Grillon et al., 1991; Olichney et al., 2002; Moreno and Kutas, 2005; Federmeier, 2009). We also evaluated the correlation between the ILF anisotropy and MMF latency. MMF reflects a process in which the infrequent sound is compared to the sensory memory trace that encodes the frequent sounds (Naatanen and Winkler, 1999). Thus, this response indicates early cognitive auditory processing. MMF is a reasonable ERF for comparing with the N400m, because they both reflect early cognitive processing at a pre-attentive level, which does not require active analysis or overt responses of the target stimuli (Holcomb, 1988; Naatanen et al., 1993; Deacon et al., 2000; Kiefer, 2002). Although MMF has been found to be sensitive to lexical elements of a presented stimulus (i.e., words elicit greater MMN amplitude than meaningless pseudo-words), it does not involve semantic processing of presented words to the same extent as N400m does (Pulvermuller et al., 2001, 2004; Pulvermuller and Shtyrov, 2006). Moreover, in our protocol, subjects were binaurally exposed to pure tones in the absence of an active task, which likely minimized the contribution of linguistic processing. It is also important to note that MMF localizes in the primary and secondary auditory cortices and right inferior frontal gyrus (Hari et al., 1984) which are not connected by the ILF. Our results showed that MMF latency did not correlate with the anisotropy of the ILF. This suggests that the role of the ILF is specific to visual semantic processing when compared to early auditory cognitive processing measured by MMF, and that this association is not a phenomenon due to a subject’s general cognitive processing ability or efficiency. In our study, we have combined MEG mapping and DTI analysis; we recorded N400m using MEG for visual semantic processing efficiency and acquired the ILF anisotropy using DTI for structural connectivity. Although preliminary, because our sample size was small, linking these two techniques provided a powerful and effective tool, because it allowed us to assess the relationship between functional and structural components of visual semantic processing in a non-invasive manner. While our task was suitable for group analysis to compare differences between “related” and “unrelated” conditions, as it did allow us to observe that the mean FA of the ILF negatively correlated with N400m latency and amplitude, it did not significantly correlate with reaction time, and accuracy. Thus, our task for measuring visual semantic processing may not be the best design for amplifying the inter-individual differences in processing efficiency. In follow-up studies it may be more effective to use more complex visual lexical tasks, which require subjects to actively engage in cognitive processing or presented words. In summary, we have demonstrated that efficient visual semantic processing assessed by N400m is correlated with high ILF anisotropy. Our findings support the view that individual differences in white matter tract integrity reflects individual ability and efficiency of cognitive functions, because the white matter tracts connect and transmit information between brain areas in a functional network (Johansen-Berg, 2010). Combined, MEG and DTI techniques can identify key white matter tracts that contribute to functional networks and contribute to our understanding of how they affect function.

It is important to highlight that these observations should take into account the following limitations. Sample size was small and there was a selection bias of highly educated individuals volunteering from campus. This however, might further ensure that our approach to measure FA as a degree of microstructural organization could have a counterbalancing net result in our analysis for the very remote possibility of changes/abnormalities that could be revealed in diffusivity parameters. Finally, although the chosen methodology, to extract ILF FA from a VOI in the stereotaxic space, being potentially influenced by effects of interpolation, we still think that our approach remains less sensitive to the interference of gray matter voxels surrounding the VOI, as if alternatively native space would be used for this extraction. Moreover, as the results of our pilot study could enable further comparisons between healthy subjects and patients, the use of such an approach makes it feasible to perform these analyses in a time efficient and accurate manner.

Age-related differences have been reported on white matter integrity and N400. Bennett et al. (2010, 2011) have shown the age-related decrease in white matter integrity measured by FA, axial diffusivity, and radial diffusivity using DTI. The authors compared two age groups: 14 undergraduate students (18–20 years old) and 14 older adults (63–72 years old) whose neuropsychological test performance was significantly declined. Our study is based on a group of healthy participants with a fairly narrow age range and no age-dependent differences in behavioral performance. The participants in our study may be considered as young adults who are yet to show deteriorations in white matter integrity. Thus, despite the small sample size, it is difficult to assume that age-dependent microstructure deterioration may interfere with our current findings in the association between the ILF and visual semantic processing.

Kutas and Iragui (1998) showed age-related changes regarding N400. Along with a significant difference in N400 amplitude in response to visually presented congruent and incongruent target words, they showed a linear decrease in N400 amplitude (0.05–0.09 mV per year, *r* = 0.40) and a linear increase in N400 peak latency (1.5–2.1 ms/year, *r* = 0.60) with age. Even if such age-related change could be applicable to our study group aged 18–36 years old, this would not affect our findings. Our study aim was to investigate the structure-function relationship regarding semantic processing and our data may rather serve as an additional evidence for structure-function relationships, potentially connecting the gap so far existing between age-dependent functional decline and age-dependent structural deterioration.

## Conclusion

We examined the role of the ILF in language comprehension using DTI and MEG. Through a semantic categorization decision task in which subjects assessed the meaning of words, we measured N400m latency and amplitude as indices for visual semantic processing efficiency using MEG. We used FA as the index for the anisotropy of the ILF in DTI and reaction time and accuracy of the responses as behavioral indices for performance efficacy. Our results demonstrate that the ILF’s role in semantic processing is not restricted to the previously described auditory modality, but also encompasses visual semantic processing. Our study further supports and extends current understanding on the ILF’s function in visual processing and language comprehension (Saur et al., 2008; Agosta et al., 2010; Wong et al., 2011; Ortibus et al., 2012; Taddei et al., 2012).

## Ethics Statement

This study was carried out in accordance with the recommendations of the Montreal Neurological Institute Research Ethics Board with written informed consent from all subjects. All subjects gave written informed consent in accordance with the Declaration of Helsinki. The protocol was approved by the Montreal Neurological Institute Research Ethics Board.

## Author Contributions

JS acquired and analyzed the data and drafted, edited and corrected the manuscript. JR analyzed the data, and drafted, edited and corrected the manuscript. RC analyzed the data, and edited and corrected the manuscript. PJ, CG, and PR-N participated in the design of the study, data analysis, and corrected the manuscript. DK participated in the design of the study and corrected the manuscript. EK participated in the design of the study, data acquisition, data analysis, drafted and corrected the manuscript.

## Conflict of Interest Statement

The authors declare that the research was conducted in the absence of any commercial or financial relationships that could be construed as a potential conflict of interest.

## Footnotes

1 http://lbam.med.jhmi.edu
